# Outcomes in Pregnant Women with Valvular Heart Disease from Portuguese-Speaking African Countries Treated in Portugal through an International Agreement of Health Cooperation

**DOI:** 10.5334/gh.1183

**Published:** 2023-02-13

**Authors:** Vera Vaz Ferreira, André Viveiros Monteiro, Rita Ilhão Moreira, Marta Plancha, Ana Isabel Machado, Alexandra Castelo, Pedro Garcia Brás, Tânia Branco Mano, Maria José Alves, Boban Thomas, Rui Cruz Ferreira, Lino Patrício

**Affiliations:** 1 Department of Cardiology, Hospital de Santa Marta, Centro Hospitalar Universitário de Lisboa Central, Lisbon, PT; 2 Department of Obstetrics and Gynecology, Maternidade Alfredo da Costa, Centro Hospitalar Universitário de Lisboa Central, Lisbon, PT; 3 The Heart Centre, Hospital Cruz Vermelha Portuguesa, Lisbon, PT; 4 Cardiology Department and CRIA/Cerebro-Cardiovascular Responsability Centre, Hospital do Espírito Santo, Évora, PT

**Keywords:** valvular heart disease, mechanical heart valves, pregnancy outcomes, Portuguese-speaking African countries, intergovernmental health agreements

## Abstract

**Aims:**

We performed a clinical audit of maternal and fetal outcomes in pregnant women with valvular heart disease (VHD) from Portuguese-speaking African countries who were transferred for their care, during a twenty-year period, through a memorandum of agreement of international cooperation.

**Methods and results:**

A retrospective analysis of 81 pregnancies in 45 patients with VHD (median age 24, interquartile range 22–29 years) from 2000 to 2020 was performed. The main outcome measures were maternal cardiovascular and fetal outcomes. History of rheumatic heart disease was present in 60 (74.1%) pregnancies. Most were in New York Heart Association (NYHA) functional class I or II; at the first evaluation, 35 (43.2%) were on cardiac medication and 49 (60.5%) were anticoagulated. Forty-eight pregnancies had at least one valvular prosthesis, including 38 mechanical heart valves. During pregnancy, deterioration in NYHA functional class occurred in 35 (42.0%), and eight (9.9%) patients required initiation or intensified cardiac medication. Mechanical valve thrombosis complicated four (4.9%) pregnancies, all cases on heparin, and resulted in one maternal death. Haemorrhagic complications happened in 7 (8.6%) anticoagulated patients, in the immediate postpartum or puerperal period. The 81 pregnancies resulted in 56 (69.1%) live births, while miscarriage and fetal malformations occurred in 19 (23.5%) and 12 (14.8%) pregnancies, respectively. In multivariate analysis, vitamin K antagonist therapy was the only independent predictor of an unsuccessful pregnancy (p = 0.048).

**Conclusion:**

In a high-income country, successful pregnancy was possible with low rate of maternal events in women with VHD transferred from five low-middle income countries in Africa. The use of anticoagulation with a vitamin K antagonist was associated with an unsuccessful pregnancy.

## Introduction

Recent advances in surgical techniques and the medical management of valvular heart disease (VHD) in developed countries have permitted young patients to have an improved prognosis and quality of life, with a significant number of women having successful pregnancies [1]. VHD is primarily caused by rheumatic heart disease (RHD) in low-middle income countries (LMICs), and sub-Saharan Africa bears a significant portion of the global burden of the disease [2]. Most patients with VHD in Africa do not avail themselves of required surgery despite being symptomatic because of lack of cardiac surgical services [3]. Pregnant women with VHD in African countries have a high maternal morbidity and mortality due to the reduced accessibility to health care and the scarcity of skilled health care professionals [4]. Patients with VHD are a particular high-risk group in general, since the physiologic haemodynamic changes in pregnancy increase the risk of heart failure and arrhythmias, with potential adverse impact on both maternal and fetal outcomes [5,6,7,8,9]. Our group has previously reported on the data over a 10-year period with VHD and pregnancy [10]. For the surgical management of these patients in Africa, generally three models have existed. The first was the referral of some patients to high-income countries (HIC) through agreements or various channels. The second has been the surgical repair of these lesions by visiting teams to Africa at regular intervals [11]. The third is the development of local cardiac surgical capacity [12]. However, very little data exists in the public domain regarding outcomes in pregnant women with VHD followed in a HIC after they had been referred specifically for management of their condition.

Through a health agreement of international cooperation, Portugal provides medical and surgical treatments of cardiac disease for patients from Portuguese-speaking African countries, that include Angola, Mozambique, Guinea-Bissau, Cape Verde, and São Tomé and Príncipe. In 1992, these countries formed an interstate organization known as *Países Africanos de Língua Oficial Portuguesa* (PALOP). At least three countries (Guinea-Bissau, Cape Verde, São Tomé and Príncipe) have no facilities for either cardiac catheterization or cardiac surgery. Under this agreement, annually about 1000 patients with diverse medical and surgical conditions have access to the tax-funded universal access health system of Portugal—the Serviço Nacional de Saúde (SNS) [13]. These patients can be referred to various cardiology/cardiac surgery departments in Portugal for evaluation and management of mainly severe VHD, and our departments form part of the network that provides this service.

We performed a clinical audit of pregnant women with VHD from five African nations, followed at our tertiary centre, during a twenty-year period, evaluating maternal cardiovascular and pregnancy outcomes. Our aim was to determine if rigorous follow-up of patients with VHD, which is difficult in the LMIC that the patients originated from (including those with mechanical heart valve (MHV) on anticoagulation), would guarantee good maternal and fetal outcomes.

## Materials and methods

### Participants and Eligibility Criteria

**Figure 1:**
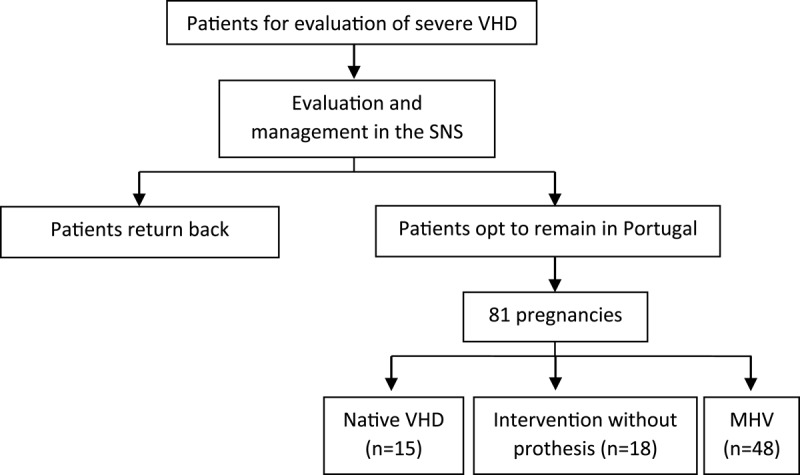
Patient trajectory in our cohort.

We conducted a retrospective clinical audit of all pregnant women with VHD from PALOP who had been referred to a tertiary institution for management over a 20-year period between 2001 and 2020. All the patients were first referred to our centre for the management of VHD and subsequently stayed behind in Portugal of their own volition (Figure 1). Only patients with moderate or severe VHD were included. This study (INV 333) was conducted according to the Declaration of Helsinki and was approved by the Ethics Committee of Centro Hospitalar Universitário de Lisboa Central. Care of the patients during the pre-conception phase, pregnancy and delivery was provided in a cardio-obstetrics unit, involving cardiologists, obstetricians, anesthesiologists, internists and cardiac surgeons. All patients were delivered at our centre and evaluated by a paediatrician at the time of delivery and on the fifth day of life. No patients were lost to follow up.

The data collected included demographic, obstetric, neonatal and cardiac details obtained at the time of the first visit, and also during and after pregnancy. For patients with more than one pregnancy, each gestation was considered a new dataset. Recorded cardiovascular variables included previous cardiac surgery or percutaneous intervention, history of cardiac complications, New York Heart Association (NYHA) functional class, left ventricular systolic function, and ongoing medications at the time of conception, including anticoagulation. Vitamin K antagonist (VKA) and heparin-based regimes were used to manage anticoagulation. Most VKA to low molecular weight heparin (LMWH)-based regimen shifts occurred because of high VKA dosages required for effective International Normalized Ratio (INR, >5 mg in warfarin and >2 mg in acenocumarol). The INR was maintained between 2.5 and 3.5 in patients with MHV. Patients were followed at least monthly during the entire pregnancy and weekly after 34 weeks of gestation. In patients under VKA in the third trimester, a switch to LMWH (1 mg/kg every 12 hours) was usually performed around 36 weeks. One to two weeks later, patients were hospitalized for elective delivery and LMWH was replaced by unfractionated heparin (UFH) for at least a 36-hour period. Labour was induced and type of delivery was chosen according to obstetrical indications usually. UFH was discontinued 4–6 hours before the predicted expulsive stage of labour. After delivery and adequate haemostasis, anticoagulation with LMWH or UFH was restarted approximately in the first 6–12 hours in an individualized manner, according to the type of MHV and the risk of thromboembolism and bleeding in each patient.

During pregnancy information on use and changes in anticoagulant therapy, cardiac complications, including death, worsening of heart failure (including acute pulmonary oedema, arrhythmias, need to initiate appropriate treatment, cardiac interventions, thromboembolic and haemorrhagic), and neonatal complications were recorded. Neonatal events included premature labour (spontaneous onset of labour at <37 weeks’ gestation), small for gestational age (SGA; i.e., birth weight <10th percentile), miscarriages (until 23 weeks of pregnancy), still birth (after 23 weeks of pregnancy), neonatal death (within the first 28 days after birth), and congenital malformations of all causes, including warfarin embryopathy. Unsuccessful pregnancy in this study was defined as miscarriages, medical termination of the pregnancy before 23 weeks and still births.

### Statistical analysis

Statistical analyses were performed using SPSS software (v. 25; SPSS Inc., IBM). Data were expressed as mean ± SD or median (interquartile range [IQR]) for continuous variables and as frequencies and percentages for categorical variables. The data distribution was tested for normality using the Kolmogorov-Smirnov test or Shapiro-Wilks test, as appropriate. Univariate association of potential categorical predictors and the maternal or pregnancy outcomes was tested using the Chi square or Fisher’s exact test as appropriate whereas that between numerical covariates and the above outcomes was tested using independent sample t-test or Mann-Whitney U test, as appropriate.

Predictors showing a p-value of less than 0.05 on univariate analysis were entered into binary logistic regression analysis to control for confounders and determine independent predictors of unsuccessful pregnancy as the dependent variable. Clinical characteristics, baseline echocardiographic parameters and anticoagulation therapy were independent variables. All statistical tests were two-sided, and a p-value ≤0.05 was statistically significant.

## Results

### Baseline demographic and clinical characteristics

Over the 20-year study period, 81 pregnancies in 45 patients with VHD were studied. Baseline demographic and clinical details are listed in Table 1.

**Table 1:** Baseline characteristics of the patients.


	N = 81

**Age (years), median (interquartile range)**	24 (22–28.5)

**Multiparous, n (%)**	34 (42.0%)

**Hypertension, n (%)**	4 (4.9%)

**Smoking habits, n (%)**	2 (2.5%)

**Congenital heart disease, n (%)**	9 (11.1%)

**Rheumatic fever, n (%)**	60 (74.1%)

**Previous cardiovascular medication, n (%)**	35 (43.2%)

**Impaired left ventricular ejection fraction, n (%)**	10 (12.3%)

**NYHA class III or IV, n (%)**	17 (21.0%)

**Previous arrhythmic event, n (%)**	10 (12.3%)

**Previous stroke, n (%)**	7 (8.6%)

**Previous infectious endocarditis, n (%)**	5 (6.2%)

**Anticoagulation, n (%)**	49 (60.5%)

Warfarin	22 (27.2%)

Acenocoumarol	27 (33.3%)


NYHA, New York Heart Association.

Twenty-four women had one, 13 women had two, four women had three, two women had four, one woman had five and another woman had six pregnancies in our unit during the study period (Table S1). The median maternal age at pregnancy was 24 years (IQR 22–29) and 34 (42.0%) patients were multiparous. A self-reported or referring physician’s history of rheumatic fever was noted in 60 (74.0%) pregnancies. At the start of pregnancy, 17 patients (21.0%) were in NYHA class III or IV and 10 (12.3%) had impaired left ventricular ejection fraction. Untreated valvular conditions, previous surgical or percutaneous correction before conception, and a valvular prosthesis, were present in 15 (18.5%), 18 (22.2%) and 48 (59.3%) pregnancies, respectively. As depicted in Table 2, isolated involvement of mitral valve was the most frequent in 54 (66.7%), followed by mitral and aortic valve involvement in 12 (14.8%) pregnancies.

**Table 2:** Valvular heart disease characterization.


	N = 81

**Cardiac valve involved, n (%)**	

Mitral	54 (66.7%)

Mitral and aortic	12 (14.8%)

Mitral and tricuspid	7 (8.6%)

Aortic	5 (6.2%)

Pulmonary	3 (3.7%)

**Native valvular disease, n (%)**	15 (18.5%)

Mitral regurgitation	5 (6.2%)

Aortic and mitral regurgitation	4 (4.9%)

Mitral valve prolapse with regurgitation	3 (3.7%)

Aortic regurgitation	2 (2.5%)

Moderate mitral stenosis	1 (3.3%)

**Previous surgical or percutaneous correction before conception (without prosthesis), n (%)**	18 (22.2%)

Mitral stenosis/regurgitation	13 (16.0%)

Pulmonary stenosis	3 (3.7%)

Aortic stenosis	2 (2.5%)

**Valvular prosthesis, n (%)**	48 (59.3%)

Mechanical prosthesis	38 (46.9%)

Mitral	43 (53.1%)

Aortic	8 (9.9%)


### Anticoagulation

Forty-nine (60.5%) were anticoagulated before pregnancy, 22 (27.2%) with warfarin and 27 (33.3%) with acenocoumarin. MHV was the indication for anticoagulant therapy in 38 pregnancies (77.6%), followed by atrial fibrillation and a recent venous thrombo-embolic event.

**Figure 2:**
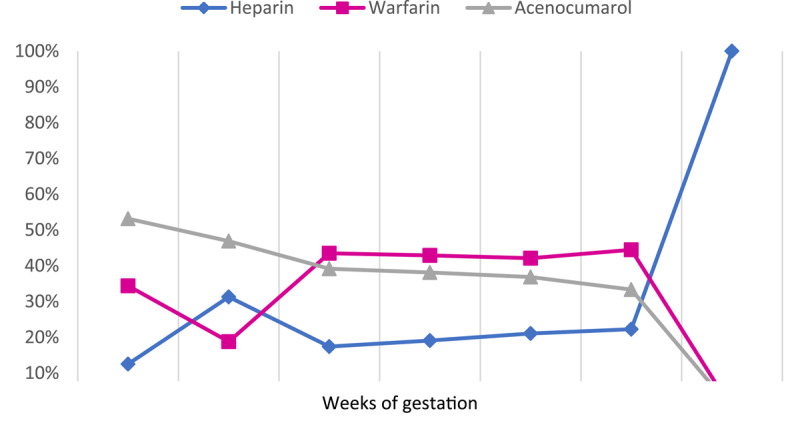
Distribution of anticoagulant treatment according to the weeks of gestation in patients with MHV.

Thirty-four pregnancies (69.4%) were maintained on the same regimen, VKA, during the three trimesters of pregnancy and 15 (30.6%) were switched to a heparin-based regimen during a part of or the entire pregnancy. Nine (18.4%) received heparin subcutaneously between 6–12 weeks and six (12.2%) continued throughout pregnancy (Figure 2).

### Clinical outcomes

#### Maternal outcomes

Details of maternal outcomes are summarized in Table 3. A deterioration in NYHA class occurred in 35 (43.0%) pregnancies and eight (9.9%) required initiation or intensification of cardiac medications. Acute pulmonary oedema (APE) occurred in four (4.9%) pregnancies (Table S2), during the third trimester of pregnancy and in patients with baseline NYHA class III or IV.

**Table 3:** Obstetric and fetal outcomes.


	N = 81

**Maternal outcomes**	

NYHA class worsening, n (%)	35 (42.0%)

Need to initiate/intensify cardiac medication, n (%)	8 (9.9%)

Acute pulmonary oedema, n (%)	4 (4.9%)

Haemorrhagic complications, n (%)	7 (8.6%)

Thrombotic complications, n (%)	4 (4.9%)

All-cause mortality, n (%)	1 (1.2%)

**Fetal outcomes**	

Live birth, n (%)	56 (69.1%)

Miscarriages, n (%)	19 (23.5%)

Stillbirth, n (%)	6 (7.4%)

Fetal malformations, n (%)	12 (14.8%)

Gestational age at delivery (weeks), mean ± SD	35.4 ± 6.9

Weight, g	3026.4 ± 471.4

Apgar index > 7 at 5^th^ minute, n (%)	51 (91.1%)

Labour, n (%)	60 (74.1%)

Labour type	

Vaginal	43 (71.7%)

Cesarean	17 (28.3%)

Labour indication	

Obstretic	56 (93.3%)

Cardiac	4 (6.6%)


NYHA, New York Heart Association.

There were four prosthetic mitral valve thromboses (Table S3) in the first trimester, all in patients receiving LMWH. One of the cases resulted in maternal death despite emergent surgical intervention for occlusive mitral thrombus and cardiogenic shock. This woman refused VKA during the first trimester owing to a previous history of warfarin embryopathy and had a therapeutic anti-XA in the last evaluation prior to the event. The other three patients were treated successfully with intravenous UFH, leading to two live births and one miscarriage.

Bleeding events requiring transfusion or surgical revision occurred in seven pregnancies (8.6%) and included four anterior abdominal wall hematomas and three peritoneal hematomas located in the retro-uterine pouch. They happened in immediate postpartum or puerperal period in four patients on warfarin, two on LMWH and one on acenocoumarol.

We do not have complete data on contraceptive methods used by each patient in the study. The commonest methods used in our cohort were combined hormonal contraceptive (CHC) pill and long-acting reversible contraception, including implants and intrauterine devices. Some had also undergone permanent methods, like tubal sterilisation after the desired number of successful pregnancies.

#### Obstetric and fetal outcomes

Details of obstetric and neonatal outcomes are listed in Table 3. The 81 pregnancies resulted in 60 labour attempts with 56 (69.1%) live births, with a mean birth weight of live-born neonates of 3026 ± 471 g. Delivery by caesarean section (CS) was performed in 17 (28.3%), four of them (6.6%) due to a maternal cardiac indication.

Miscarriages and stillbirth occurred in 19 (23.5%) and six (7.4%) pregnancies, respectively. Fetal malformations were found in 12 (14.8%) newborns, including four cases of warfarin embryopathy.

The median individual success ratio (number of live births/number of total pregnancies for each woman) was 0.5 (IQR 0.5–1.0). A successful pregnancy occurred more frequently in patients on heparin compared with those on VKA (84.6 vs 44.4%, p = 0.025). No associations between fetal outcomes and VKA dosage were found in our sample.

In univariate analysis, the presence of valvular protheses (OR 8.46, 95%CI 2.27–31.54, p = 0.001), and anticoagulation (OR 13.27, 95%CI 2.85–61.73, p = 0.001) were related to unsuccessful pregnancy. Maternal outcomes did not significantly differ in multiparous compared with nulliparous women. In multivariate analysis, VKA therapy was the only independent predictor of unsuccessful pregnancy (OR 7.20, 95%CI 1.02–50.94, p = 0.048).

## Discussion

In this retrospective descriptive clinical audit conducted at our tertiary centre, we reviewed the outcomes of 81 pregnancies in patients with severe VHD, initially transferred from five Portuguese-speaking African countries, but mainly Cape Verde, São Tomé, and Príncipe and Guinea-Bissau for management of their VHD as these countries have no cardiac surgical facilities.

In this cohort, the use of VKA was the single most important predictor of an unsuccessful pregnancy. The rate of live birth was higher in patients on heparin compared with those on VKA. Mirroring other experiences [14,15,16], in four pregnancies with a MHV an acute valve thrombosis occurred, all in patients on LMWH. The patient who died had a therapeutic anti-Xa level in the visit preceding her demise. Significant bleeding events occurred in 8.6% of the pregnancies, all anticoagulated, in immediate postpartum or puerperal period. The patients in our cohort were managed according to current international guidelines. The ESC [17] and AHA/ACA guidelines [18] recommend the use of VKAs until pregnancy is achieved. Given the known dose-dependent teratogenicity, continuation of VKAs should be considered during the first trimester in patients with a low daily dosing of a VKA (warfarin <5 mg/day or acenocoumarol <2 mg/day), ideally in a shared decision-making paradigm. Guidelines also recommend the sequential anticoagulation with heparin from 6–12 weeks, under strict monitoring, primarily in those who need a higher daily dose of VKA. Both guidelines recommend that VKAs should be used during the second and third trimesters, irrespective of dose, until approximately 36 weeks’ gestation.

Real world data back up the guidelines with some important nuances. In a study by Mazibuko and colleagues from South Africa, 29 (50%) patients were on warfarin ≤ 5 mg in the first trimester and no congenital fetal anomalies occurred, replicating reports from India, Oman and Lebanon [16,19,20]. In the meta-analysis by D’Souza et al. [21], the often-quoted ceiling dose of 5 mg was established for warfarin beyond which the fetal adverse events increased. However, the ROPAC registry [14] could not confirm this margin of safety. Fetal complications can occur even with daily doses < 5 mg [22,23]. VKAs come packaged with their own logistical constraints particularly in LMICs. In Ethiopia, Chalachew et al. [24] have shown that the educational level of primary school or less, long distance from follow-up medical facility, infrequent check-up visits and unreliable source of free warfarin supply were associated with sub-optimal control of INR. These conditions were also noted in Nigeria, the most populous nation in Africa, making a VKA a difficult choice for a patient with a MHV that can portend a disastrous outcome [25].

LMWH is often advocated as an option in HIC for the first trimester. Under rigorous monitoring, some institutions even have protocols that use LMWH throughout pregnancy [26]. Although the use of LMWH with peak and trough levels of anti-Xa levels monitoring seems a promising approach, the risk of MHV thrombosis is still a heavy burden. A recent study from India found that healthcare access, costs, compliance with parenteral therapy, limited training in administration and availability of monitoring techniques were significant challenges [27]. The risk of valve thrombosis with LMWH persists, even when with therapeutic anti-factor Xa activity levels [27,28].

The newer oral anticoagulants are not an option in those with MHV and all the studies (including iNVICTUS [29]) excluded pregnant patients. This leaves VKAs as the best option to protect the mother albeit at risks of fetal embryopathy.

In our study, 42% of pregnancies occurred in multiparous women and four patients were responsible for approximately 25% of the total pregnancies. However, in each woman, about half of pregnancies were successful. This underscores the need for multiple pregnancies to obtain favourable fetal outcomes, mainly when anticoagulated. Notwithstanding this fact, the increased number of pregnancies per woman was not associated with major maternal adverse cardiac events. The majority (79.0%) were in NYHA functional class I or II at the onset of gestation. A symptomatic deterioration in NYHA functional class was the most important cardiac complication (42.0%) and 9.9% needed to initiate or intensify cardiac medication. APE was rare (4.9%) and effectively reverted with medical therapy. The relative paucity of APE in our cohort could be due to early pregnancy assessment, adequate medical therapy and the absence of significant number of patients with untreated mitral stenosis which is the commonest cause of APE in LMIC. In the absence of severely regurgitant lesions, APE is rare. Our findings are in line with the CARPREG II study where a decrease in the incidence of APE was documented over two different time periods [30].

In our study, the rate of live births was 69.1%, miscarriage and stillbirth occurred in 23.5% and 7.4% of pregnancies, respectively. A CS was performed in 28.3%, a higher value compared to that in the normal population as recorded in an European registry [31], although an absolute cardiac indication was present in only 6.6%. However, this may be related to clinician preference as seen in a comparison between two European obstetric cardiac centres, where the rate of CS was 74% in one centre and 28% in another for patients with MHV, demonstrating varying practice patterns [32].

In our experience, management of these patients with a MHV is resource-intensive and, every attempt at valve repair, although particularly difficult, should be attempted. Truth be told, the paucity of these patients on the regular cardiac surgical list of a surgeon in a developed country precludes the attainment of adequate expertise, as evidenced in a Korean study where just two cases were performed per surgeon per year [33]. Valve repair provides the opportunity for women to possibly go through their pregnancy without anticoagulation and its complications. In most cases, though, a durable repair—even in expert hands—lasts for approximately a little more than a decade [34]. Bioprosthetic valves are prone to rapid structural deterioration, especially during pregnancy, as described in a study from Rwanda [35]. Patients may present late, precluding repair and cardiac surgical facilities are sparse in Africa.

One of the biggest contributions HIC can make is in the training of professionals from African countries. Tefera et al. [36] reported the experiences of local surgeons and the visiting surgeons in Ethiopia. Local surgeons mentioned few cases performed under supervision and the lack of sufficient training provided by visiting surgeons, who also considered it an ineffective model for skill transfer. Improvement of cardiac surgical services can be possible through collabouration but will only be successful if real efforts are made to train local personnel and introduce sustainability measures [12].

All the patients had cardiac surgery in Portugal and continued to live here of their own volition with a relatively good functional status. Concerns such as access to medications, medical care in the case of complications, the ability to make independent health choices, and the understanding that better obstetric care will be available within a multi-disciplinary unit convinced some women to remain behind in Portugal. Traditional beliefs and cultural contexts around childbearing can be discussed, allowing women to make informed decisions regarding their care and contraception. Choosing a method of contraception for women with VHD requires consideration of safety, effectiveness, patient preference and the risk of an unplanned pregnancy. The use of the CHC has an increased risk of thromboembolism which is particularly relevant in those with MHVs.

These factors call for increased coverage of interventions to control and manage RHD to accelerate progress towards its eradication in African countries [37]. Despite decades of clinical experience with penicillin in streptococcal pharyngitis to prevent rheumatic fever and RHD, rates of untreated pharyngitis remain very high among school-aged children in most LMIC [38]. Clinical decision-models that are simple can help prevent rheumatic fever and RHD in the countries that our patients were referred from and represent the low hanging fruit that should be plucked easily [39].

## Limitations

This is an analysis of data from a 20-year period. Some elements were missing from the earliest patients in the cohort. History of rheumatic fever was either self-reported or communicated by referring physician implying limitations in the fidelity of this information. In our series, no arrhythmic events occurred during pregnancy. However, we cannot exclude the possibility that these patients presented to other hospitals in the SNS network and electronic health platforms with access to the limited information across the different networks became available only recently. Most patients in this cohort received an MHV, which could also indicate the difficulty of repair in RHD, late referral and the relative inexperience of cardiac surgeons in HIC for this pathology. It is possible that better fetal outcomes would have been possible in this same cohort if repair was the primary approach. However, we emphasise that in our experience patients are often referred very late from these countries, precluding a durable repair. The health care systems of some of these nations are rudimentary for cardiac services and diagnosis is often delayed due to lack of imaging. The process of referral is also very convoluted in those countries with a panoply of bureaucratic procedures hindering timely transfer of patients. The sample size of the study population reflects the limitations of a single-centre experience, and a potential selection bias cannot be excluded. The confidence intervals in the univariate and multivariate analysis are wide due the small number of patients.

## Conclusions

Pregnant women with VHD from five Portuguese-speaking African countries managed in a tertiary care centre in Portugal, through an intergovernmental agreement, can achieve successful pregnancies with low maternal cardiovascular complications. Significant fetal complications arise, despite vigilant control. VKA therapy was the only independent predictor of an unsuccessful pregnancy. Despite the relative success of such a care model, its applicability is limited due to logistic constraints for pregnant women with VHD in many countries of the African subcontinent. Valve repair should be pursued to hopefully avoid anticoagulation during pregnancy.

## Data Accessibility Statements

The data underlying this article are available in the article and in its online supplementary material.

## Additional File

The additional file for this article can be found as follows:

10.5334/gh.1183.s1 Supplemental Data. Tables S1 to S3.
