# Supplementary material for: Outcomes in Pregnant Women with Valvular Heart Disease from Portuguese-Speaking African Countries Treated in Portugal through an International Agreement of Health Cooperation
Source: Glob Heart. 2023 Feb 13;18(1):4. doi: 10.5334/gh.1183 (PMC9936910; doi:10.5334/gh.1183)
Supplement: Supplemental Data. — Tables S1 to S3. [file gh-18-1-1183-s1.pdf]

Supplementary Data

Table S1 – Outcomes on women with more than 3 pregnancies during follow-up

Women	Pregnancy	Age	Year	VHD	Oral anticoagulation during pregnancy	Fetal outcome	Fetal malformations	Maternal complications
**1**	1	19	2001	Mechanical mitral valve prosthesis	W	Live birth	-	Hemorrhagic
	2	21	2003		W	Miscarriage	-	-
	3	22	2004		W> LMWH (6-12th weeks) > WF	Live birth	-	-
	4	28	2009		W> LMWH (6-12th weeks) > WF	Live birth	-	-
**2**	1	23	2001	Mechanical mitral valve prosthesis	W	Stillbirth	-	-
	2	24	2002		A	Live birth	-	Prosthestic valve thrombosis
	3	35	2011		A	Miscarriage	-	Hemorrhagic
	4	36	2012		A	Live birth	-	-
**3**	1	20	2010	Mechanical mitral valve prosthesis	A	Stillbirth	VKA embryopathy	-
	2	22	2012		A	Miscarriage	-	-
	3	23	2013		A	Miscarriage	VKA embryopathy	-
	4	24	2014		A	Miscarriage	-	-
	5	26	2016		LMWH	Live birth	-	-
**4**	1	17	2001	Aortic and mitral regurgitation	No	Live birth	-	-
	2	18	2002		No	Live birth	-	-
	3	22	2006	Biologic aortic valve prothesis and mitral repair	W	Miscarriage	Neurological	-
	4	23	2007		W	Miscarriage	-	-
	5	24	2008		LMWH	Live birth	-	-
	6	32	2016		W> LMWH (6-12th weeks) > W	Miscarriage	VKA embryopathy	-

VHD: valvular heart disease; VKA: vitamin K antagonist, AC: acenocumarol, W: warfarin, LMWH: Low molecular weight heparin

Table S2 – Acute pulmonary edema

	Age	Year	Etiology	Diagnosis	VKA	Gestational trimester	Treatment	Maternal mortality	Fetal mortality
**1**	25	2002	Rheumatic	Severe mitral regurgitation	-	2st	Emergent surgery	No	Yes (still birth)
**2**	25	2005	Rheumatic	Biologic mitral valve	W	3st	Medical	No	No (caesarian)
**3**	25	2012	Rheumatic	Severe aortic regurgitation	-	2st	Medical	No	No (pre-term)
**4**	22	2019	Rheumatic	Mitral + aortic mechanical prothesis	A	1st	Emergent surgery	Yes	Yes

VKA: vitamin K antagonist, A: acenocoumarol, W: warfarin,

Table S3 – Prosthetic valve thromboses

	Age	Year	Etiology	Valve position	Valve thrombosis	Previous miscarriage	VKA	Gestational trimester	AC < 14 weeks	AC 14-36 weeks	anti-XA	AC delivery	Treatment	Maternal mortality	Fetal mortality
**1**	24	2002	Rheumatic	Mitral	Mitral	Yes	A	1st	LMWH	A	not available	UFH	UFH	No	No
**2**	22	2012	Rheumatic	Mitral+ Tricuspid	Mitral	No	W	1st	LMWH	-	unknown	-	UFH	No	Yes
**3**	27	2012	Rheumatic	Mitral	Mitral	No	A	1st	LMWH	A	therapeutic	UFH	UFH	No	No
**4**	22	2019	Rheumatic	Mitral + Aortic	Mitral	Yes	A	1st	LMWH	-	therapeutic	-	Emergent surgery	Yes	Yes

VKA: vitamin K antagonist, AC: anticoagulation, CD: cardiac disease, A: acenocumarol, W: warfarin, LMWH: Low molecular weight heparin, UFH: unfractionated heparin
